# Political Polarization and Wellbeing: Investigating Potential Intrapersonal Harm From Affective Polarization

**DOI:** 10.5334/irsp.1052

**Published:** 2025-12-01

**Authors:** Brandon McMurtrie, Anja Roemer, Michael Philipp, Ross Hebden, Matt Williams

**Affiliations:** 1 Massey University, Palmerston North, New Zealand; 2 University of Canterbury, Christchurch, New Zealand; 3 Massey University, Auckland, New Zealand

**Keywords:** affective polarization, stress, social support, health, political polarization

## Abstract

Affective polarization—antipathy towards members of one’s political out-group—may pose challenges to social cohesion and personal wellbeing. Prior studies have suggested that one’s affective polarization may cause intrapersonal harm as well as interpersonal harm. It has been associated with reduced social support, increased stress, and worse physical health. This pre-registered study investigated the intrapersonal harm of affective polarization using a six-wave longitudinal survey (N = 470). Affective polarization, social support, perceived stress, and self-rated health were measured fortnightly for three months preceding the 2024 US presidential election. Random intercept cross-lagged panel models were employed to investigate the within-person effects of affective polarization on these indicators of wellbeing. Contrary to hypotheses, none of the hypothesized cross-lagged effects were significant, suggesting that changes in affective polarization did not predict changes in social support, stress, or health. However, cross-sectional analyses did reflect past findings, showing that higher levels of affective polarization were associated with lower social support, greater stress, and worse health. We additionally found evidence for perceived stress causing moderate increases in affective polarization. Stable differences by political orientation were also observed in our sample, with liberals reporting higher affective polarization and stress, lower social support, and worse health. Despite the lack of significant effects, potentially due to limitations such as sample size and measurement constraints, our findings underscore the importance of further investigations with appropriate robust designs to clarify the relationship between affective polarization and wellbeing. These results challenge the assumption that affective polarization directly drives declines in wellbeing.

## Introduction

A wealth of research has shown that, in recent decades, affective polarization—antipathy and aversion to one’s political out-group—has been increasing between members of political-ideological groups in the United States and in other countries around the world (Boxell, Gentzkow & Shapiro, 2022; Finkel et al., 2020; Iyengar, Sood & Lelkes, 2012; Kekkonen & Ylä-Anttila, 2021; Orriols & León, 2020). People appear to increasingly dislike members of their ideological-political out-groups, being increasingly reluctant to associate or have family ties with them (Huber & Malhotra, 2017; Iyengar, Sood & Lelkes, 2012), appraising them more negatively (Carmines & Nassar, 2022), and expressing and displaying prejudice towards them (Gift & Gift, 2015; Iyengar & Westwood, 2015; Westwood et al., 2018).

Affective polarization has been shown to influence a range of phenomena and increase behaviors that are counterproductive in pluralistic societies that rely on a certain level of goodwill and cooperation between ideological groups. Experiments have shown that it politicizes and undermines ideals of political belief formation (Druckman, Peterson & Slothuus, 2013; Druckman et al., 2021) and increases belief in in-group-congruent misinformation (Jenke, 2023). Longitudinal studies suggest that it negatively impacts support for democratic norms (Kingzette et al., 2021), and it likewise harms social trust (Lee, 2022; Torcal & Thomson, 2023). Additionally, expressed prejudice based on political orientation can exceed prejudices based on race, religion, and gender (Carmines & Nassar, 2022), and multiple studies using behavioral experiments such as selection tasks and trust games have verified this (Iyengar & Westwood, 2015; Lelkes & Westwood, 2017; Westwood et al., 2018). Other experiments have shown that this animosity also affects economic decisions and willingness to cooperate with others (Engelhardt & Utych, 2020; McConnell et al., 2018), and can drive discrimination in employee selection (Gift & Gift, 2015), dating preferences (Huber & Malhotra, 2017; Nicholson et al., 2016), and even roommate selection (Shafranek, 2021).

While these findings highlight how affective polarization damages the socio-political environment and promotes prejudice toward others, some research suggests it also harms the polarized individual—negatively affecting their interpersonal relationships, stress levels, and even physical health. The present study investigated this potential for intrapersonal harm using a longitudinal survey measuring the impact of affective polarization on social support, stress, and health.

### Affective polarization, social support, stress, and health

Social support is the perception that one is a part of a social network of individuals who can provide love, care, support, and mutual assistance. It can come from relatives, romantic partners, co-workers, mentors, or other social-community ties (Taylor, 2012). Social support can help lower anxiety and depression and consistently reduces psychological distress during crises such as terror attacks or serious medical diagnoses. Social support powerfully predicts health and longevity outcomes, rivalling the influence of well-established risk factors such as smoking, blood pressure, obesity, and lack of physical activity. For a review of this research, see Taylor (2012).

Affective polarization is usually studied through the lenses of social identity theory and intergroup threat theory (Tajfel & Turner, 2001; Stephan, Ybarra & Morrison, 2009; Renström, Bäck & Carroll, 2021). Social identity theory suggests that strong in-group identification inherently creates preferences for in-group members and avoidance of out-groups, especially in the presence of so-called ‘aggravating conditions’ like contentious election periods (Brown, 2000). This aversion to the out-group drives selective association and a desire for homophily. Indeed, political affective polarization is characterized by an aversion toward—and a desire for social distance from—political out-group members (McMurtrie et al., 2024; Iyengar, Sood & Lelkes, 2012). Highly polarized individuals show particularly strong preferences for homophily in their social networks, friendships, romantic relationships, work relationships, and family ties (Arora et al., 2022; Huber & Malhotra, 2017; Iyengar, Sood & Lelkes, 2012; McPherson, Smith-Lovin & Cook, 2001), which suggests that affective polarization may constrain one’s ability to form and maintain supportive social networks, and there is some evidence for this. For example, during times of heightened polarization people are less likely to travel to visit family for holidays, and if they do, are more likely to cut visits short (Chen & Rohla, 2018; Lee, 2021). For those who live in a politically dissimilar area, affective polarization and the desire for homophily may make it more difficult to find romantic partners (Huber & Malhotra, 2017; Iyengar, Konitzer & Tedin, 2018; Nicholson et al., 2016), jobs (Gift & Gift, 2015), and is associated with lower social support (Chopik & Motyl, 2016; Panagopoulos et al., 2022). Polarization also undermines social trust, a vital component of social network formation (Lee, 2022). Political moderates find cues of strong partisanship off-putting, suggesting that polarized individuals likely drive politically disinterested others away (Klar, Krupnikov & Ryan, 2018), and people do indeed report losing meaningful relationships to politics (Smith, Hibbing & Hibbing, 2019).

Collectively, these findings suggest a theoretical mechanism whereby affective polarization undermines individuals’ ability to build and maintain robust social support networks and creates a self-reinforcing cycle of social network constriction—polarized individuals may both actively avoid out-group members and inadvertently repel potential network partners, undermining their access to diverse sources of social support. However, empirically, it is still unclear whether this is truly the case and whether it translates into lower social support among polarized partisans. Many of the studies reviewed above either do not measure affective polarization, and instead merely track behaviors during times of heightened polarization such as during elections (Chen & Rohla, 2018; Lee, 2021), or they investigate whether affective polarization and partisanship drives people to discriminate against others (Huber & Malhotra, 2017; Nicholson et al., 2016; Shafranek, 2021) with no indication of whether this translates into a loss of social support for those who are affectively polarized.

Affective polarization may also exert a direct negative effect on partisans’ mental wellbeing. Social identity theory posits that when individuals identify strongly with a group—especially when that group forms a core part of their self-concept—they become more sensitive to the presence and actions of out-groups. In competitive or ideologically charged contexts, this often leads to automatic dislike of out-group members and the perception of out-groups as symbolically or literally threatening (Stephan, Ybarra & Morrison, 2009). Such environments are hypothesized to be psychologically stressful, activating chronic vigilance for threat, anxiety about group status, and general emotional strain (Major & Schmader, 2017; Stephan, Ybarra & Morrison, 2009). Consistent with this view, individuals highly engaged in politics or socio-political discourse often report impaired wellbeing, including elevated stress, sleep disruption, and even suicidal thoughts (Smith, 2022). Affective polarization has also been linked to poorer mental health outcomes such as depression and anxiety (Fraser et al., 2022; Nayak et al., 2021; Panagopoulos et al., 2022), and stronger out-group antipathy predicts worse mental health (Smith, Hibbing & Hibbing, 2019). Moreover, affective polarization and out-group hostility are frequently accompanied by anxiety and anger—emotions that contribute to stress (Fraser et al., 2022; Hackett, Gaffney & Data, 2018). Finally, longitudinal evidence shows that stress and anxiety increased during the 2016 US election period (Hagan et al., 2020; Roche & Jacobson, 2019).

Thus, intergroup threat theory and the studies reviewed above support the idea that affective polarization increases stress. However, as with social support, the extant evidence comes from either cross-sectional studies, or from longitudinal studies which do not directly measure affective polarization but simply track indicators of stress during an election period. It may be that the stress induced during an election period is due to other factors, such as anxiety produced by a potential status loss in an election defeat or the ambient tension accompanying the election period. The current research does not allow an inference to be made as to whether being affectively polarized definitively increases one’s stress.

Theoretical models of political stress propose that affective polarization also exerts a negative effect on health. The effect is proposed to occur both indirectly, by changing citizens’ health behaviors and receptivity to public health messaging from in-group and out-group authorities, and directly, by acting as a socio-political stressor which exerts the well-known detrimental effect of stress on health (Braveman, Egerter & Williams, 2011; Nelson, 2022; Slavich, 2016). Affective polarization has its basis in cognitions of social identity threat and negative emotional reactions to out-group members, which—like other negative emotional states—has been theorized to produce physiological stress responses, including heightened cardiovascular reactivity, elevated cortisol levels, and increased allostatic load (Major & Schmader, 2017; Slavich, 2016).

The evidence supporting the relationship between affective polarization and health varies in quality and in whether it focuses on the direct or indirect effect of affective polarization on health. For example, a study by Van Bavel et al. (2024) empirically showed that polarization affected belief formation, receptivity to public health messaging, and health behaviors during the COVID-19 pandemic, which had an effect on health outcomes. They also claimed that ‘Polarization can also directly affect an individual’s health by increasing stress and feelings of isolation’ (p. 3087). Supporting this idea, Nelson (2022) found that the participants’ level of affective polarization was significantly negatively associated with a measure of self-reported health that has been shown to be a valid indicator of actual health status, and thus they asserted that affective polarization is ‘like drinking poison’ (p. 1). However, they used cross-sectional designs which are not suitable for establishing causality. Likewise, Fraser et al. (2022) found that those who feel more politically distant from the average voter in their state reported more days of both poor mental and physical health—again, a cross-sectional finding. In the same vein, Mefford et al. (2020) showed an increase in acute cardiovascular disease hospitalizations after the 2016 US presidential election, and Rosman et al. (2021) found that patients in North Carolina who were wearing cardiac monitoring devices during the election period were more likely to experience arrhythmias than at other times. However, in these studies, individuals’ affective polarization was not measured; the markers of health were simply tracked during a time of purported heightened polarization.

Thus, these studies provide only indirect theoretical support for the effect of affective polarization on health. It is not possible, based on these studies, to ascertain whether affective polarization exerts an intrapersonal negative effect on health. It is unclear in studies such as these whether it is other politics-related stress (such as a potential election loss), the polarization of those in one’s social environment, or one’s own level of affective polarization, which is related to the decline in health outcomes.

### The present study

While the findings from prior research support a theoretical argument for how affective polarization could exert a negative impact on social, mental, and physical wellbeing, such effects have not been investigated using analyses that are suitable for investigating causality. Thus, the claim that affective polarization exerts an inward harm and that ‘resentment is like drinking poison’ (Nelson, 2022, p. 508) has not yet been supported. We perform the first investigation of the intrapersonal harms of affective polarization using designs and analyses suitable for investigating causal effects.

The present six-wave longitudinal study, which ran for three months preceding the 2024 US presidential election, investigated the effect of affective polarization on social support, stress, and health, using single-indicator random intercept cross-lagged panel models (RI-CLPMs; Mulder & Hamaker, 2021). The RI-CLPM is well-suited for investigating causality because it establishes temporal precedence in estimating effects, which cannot be inferred from standard cross-sectional designs.

As in standard cross-lagged panel models, the RI-CLPM models the autoregressive effects over time, and thus controls for the carry-over effects of variables with themselves. The inclusion of the random intercepts allows the RI-CLPM to partial-out stable between-person differences and model the within-person relationships between variables more accurately, whereas the traditional cross-lagged panel model confuses interindividual associations for intraindividual processes (Hamaker, Kuiper & Grasman, 2015). The results of between-person analyses often do not reflect the actual relationships between variables *within* individuals when there are both stable traits and state level components to the variance (Hamaker, 2023). Because of this, we can assume that the cross-lagged coefficients from an RI-CLPM more accurately reflect causal relationships (Lucas, 2023), though we still cannot make strong causal claims—rather, we rely on a weaker claim akin to *Granger causality* (Bressler & Seth, 2011; Hamaker, Kuiper & Grasman, 2015). The RI-CLPM is one method of analysis which can better address questions of causality from non-experimental data.

We hypothesized that affective polarization would have a significant (*p* < .05) negative cross-lagged effect on social support (H1), a significant positive cross-lagged effect on perceived stress (H2), and a significant negative cross-lagged effect on health (H3).

## Methods

The present study received low-risk ethics approval by the first author’s institution (application ID: 4000027301), and the research was conducted in accordance with the principles outlined in the Declaration of Helsinki (WMA, 2024). The analyses and measures reported here were part of a larger research project, which included a fourth research hypothesis (H4) as seen in the pre-registration. Due to constraints of publication, word limits, and the fact that the fourth hypothesis does not directly relate to the first three hypotheses of this paper and its focus on wellbeing, the analysis of the fourth hypothesis is to be reported in a separate research paper.

### Sample size determination

The primary criterion for determining sample size was the financial constraint of the project. We aimed for a sample of 500 participants at each wave. We did, however, perform an a priori power analysis. We used the R package *powRICLPM* (Mulder, 2023; see accompanying website-app: https://jeroendmulder.github.io/powRICLPM/index.html) to perform a power analysis for a single-indicator RI-CLPM over six survey waves. The power analysis shows that with a sample size of around 500 participants, we would have a statistical power of 70% to detect standardized cross-lagged effects of 0.15, and over 80% to detect cross-lagged effects of 0.2. Given our financial constraints, we found this to be an acceptable level of power for this initial study.

A fuller description of the sample size determination process, as well as the R code for the power analysis can be seen in the pre-registration: https://osf.io/83sgc/?view_only=eaa9fbc0af614f8cb63a2b6036dfa88b.

### Participants and procedure

Participants were recruited from Prolific and were required to have listed the US in their nationality and to have self-described as either ‘Liberal’ or ‘Conservative’ on their profile. We used quota sampling to recruit an equal number of each to ensure the sample was more politically representative of the general American population. We conducted our study over a three-month pre-election period from August to October 2024. This period provided us with an opportunity to collect data at a time when the relevant constructs were likely in a state of flux, as variation in the constructs over time is a requirement for modeling change and causal effects. It also allowed us to investigate trends in these constructs seen in past election periods.

At the first wave, T1, we recruited an initial sample of 552 participants. Exclusions were applied either at the study-level, such that no data from excluded participants was used, or at the wave-level, such that only data from a particular wave was excluded. Our exclusion rules can be seen in the pre-registration (https://osf.io/83sgc/?view_only=eaa9fbc0af614f8cb63a2b6036dfa88b), and a detailed account of all exclusions can be seen in the Data Preparation file found in the OSF repository for this project (https://osf.io/d8sbw/?view_only=e3aa8c392ee44680ba494479ee39ea96). After applying the exclusion criteria at the wave and study level, we had a final sample of 470 participants (M_age_ = 44.4, SD_age_ = 14.1, 56% female, 43% male, 1% non-binary, and an even 50% liberal/conservative).

At the beginning of each survey, participants were asked to place themselves on a political orientation scale (*liberal, slightly liberal, slightly conservative, conservative*). An embedded tag was created in the survey, such that those who indicated they were liberal received a tag of ‘Target = Conservative’, and ‘Group = Liberal’, and vice versa. These were then inserted into the relevant items in the affective polarization scale, allowing the items to be tailored such that each participant was seeing the appropriate in-group and out-group labels in the items.

### Measures

The following measures were presented in random order in each survey, and the items within these measures were also presented in random order. Scores for affective polarization, perceived stress, and social support were calculated as average scores on each scale. We reported Omega total (ω_t_) reliability ranges over the course of the study. Omega total is analogous to Cronbach’s alpha but loosens the assumptions necessary for the accurate assessment of reliability using Cronbach’s alpha and has been shown to outperform it when these assumptions are violated (Dunn, Baguley & Brunsden, 2014; Flora, 2020; McNeish, 2018).

This study was part of a larger survey project, so a small number of other measures were also administered in the survey but are not described here, though the project as a whole is described in the pre-registration.

#### Affective polarization

Affective polarization was measured using the affective polarization scale (APS; McMurtrie et al., 2024). This scale (ω_t_ = 0.96–0.98 across waves) was composed of 15 items measuring Social Distance (*I try to avoid socialising with [out-group]*), Aversion (*[out-group] are immoral*), and Incivility (*I like to make [out-group] angry*). Responses were collected on a 7-point Likert scale (*strongly disagree* to *strongly agree*, with a neutral midpoint, *neither agree nor disagree*).

#### Social support

Social support was measured using the 24-item Social Provisions Scale (SPS; Curtrona & Russel, 1987). The scale (ω_t_ = 0.96–0.97) contained items measuring social support in the form of practical help, informational support, emotional support, social integration, esteem support, and providing support. Items such as ‘There are people I can depend on to help me if I really need it’ and ‘If something went wrong, no one would come to my assistance’, are measured on a 4-point scale (*strongly disagree, disagree, agree, strongly agree*). Items 2, 3, 6, 9, 10, 14, 15, 18, 19, 21, 22, and 24 were reverse coded.

#### Stress

Perceived stress was measured using the Perceived Stress Scale-10 (PSS-10; Cohen & Williamson, 1988). This scale (ω_t_ = 0.92–0.95) contains 10 items, with responses on a 5-point scale (*never* to *often)*. It is a commonly used measure of perceived stress (Lee, 2012). It contains items such as ‘In the last two weeks, how often have you been upset because of something that happened unexpectedly?’ and ‘In the last two weeks, how often have you felt that you were unable to control the important things in your life?’ Items 4, 5, 7, 8 were reverse coded.

HealthHealth status was measured using a single item: ‘Would you say that in general your health is currently poor, fair, good, very good, or excellent?’ This item has been used widely in many different countries and languages and has been shown to be a valid indicator of general health and a good predictor of mortality (Jylhä, 2009; Sturgis et al., 2001). This measure is ideal for the purposes of our study, as we have no hypotheses concerning what aspect of health is affected by affective polarization and are not attempting to predict or measure specific health disorders. The short, single item of general self-rated health is comprehensive and non-specific, capturing a general sense of one’s current health status.

### Statistical analyses

**Figure 1:**
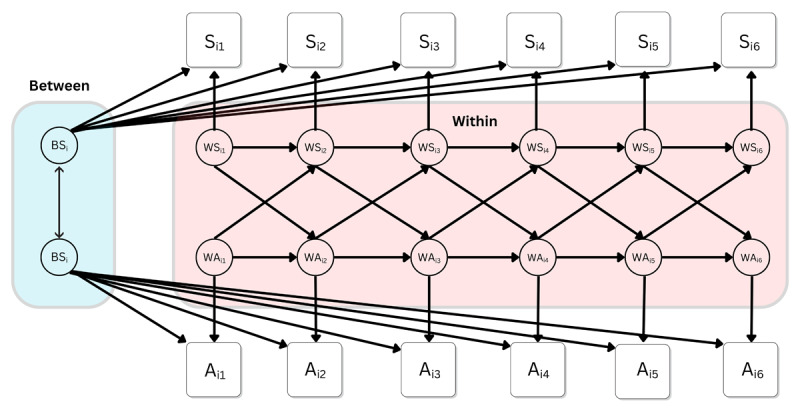
The RI-CLPM model-structure used in the present study, depicting the cross-lagged relationship between stress and affective polarization over six waves. Note: S_i1_ represents the participants’ stress score at time T1, and so on to S_i5_ at time T5; A_i1_ represents the participants’ affective polarization score at time T1, and so on to S_i5_ at time T5. BS_i_ represents the participants’ random intercept, which captures their time-invariant deviation from the grand means, or the stable trait-like component. The within components (W) at each time represent the differences between participants’ observed score and their expected score based on their random intercept and the grand means.

We used single-indicator RI-CLPMs to investigate our hypotheses. As outlined in our pre-registration, we planned to run multiple-indicator models, if possible, but were to switch to single-indicator models if the multiple-indicator models encountered errors and failures, which they did. All multiple-indicator models encountered errors such as perpetual rendering, failure to converge on a solution, and/or matrices which were not positive definite. Thus, we ran single-indicator models using the average scores at each time point, as laid out in our pre-registration. These RI-CLPMs followed the protocol of Mulder & Hamaker (2021), in which random intercepts were created as a latent variable of the repeated measures with factor loadings fixed to 1, and within-person components at each time point were the individuals’ score at that time point. The structural relations between these constructs were then laid out according to the standard RI-CLPM method as described by Mulder and Hamaker (2021). The model is visualized in Figure 1.

For all models, we constrained the autoregressive and cross-lagged coefficients to be constant over time. These assumptions were applied to the model for ease of interpretability, and are not themselves subject to testing, though our pre-registered model-fit criteria would indicate whether the model constraints and identification can be deemed appropriate. The full results of all unconstrained models can be seen in the results output in the OSF. Analyses were performed in R, version 4.3.2 (R Core team, 2023). We used robust full information maximum likelihood (MLR) estimation in the R package *lavaan*.

## Results

The full data cleaning, exclusions, and merging process from our six surveys can be seen in the Data Preparation file in our OSF. The final data set used in this study, as well as full analysis code and output can also be found there: https://osf.io/d8sbw/?view_only=e3aa8c392ee44680ba494479ee39ea96.

Descriptive statistics, including means, standard deviations, and intraclass coefficients (ICCs) for all variables in the study can be seen in Table 1. There is no clear overall trend in our constructs over the course of our study in the lead-up to the presidential election, and sample statistics for our constructs are surprisingly stable over time. Average affective polarization was below the neutral midpoint, 4, in our sample across all waves. Average perceived stress, which asks participants to report frequency of experienced symptoms, was between a response of 2 ‘almost never’ and 3 ‘sometimes’. The social support scale had no neutral midpoint, and average responses were consistently between ‘agree’ and ‘strongly agree’. The average health response in our sample was slightly above 3, which indicates a response of ‘good’ health.

**Table 1:** Descriptive statistics for all measures in the present study across the 6 survey waves (T1–T6).


	T1 M (SD)	T2 M (SD)	T3 M (SD)	T4 M (SD)	T5 M (SD)	T6 M (SD)	ICC

Affective polarization	3.5 (1.4)	3.4 (1.5)	3.6 (1.5)	3.5 (1.5)	3.5 (1.5)	3.6 (1.5)	0.86

Social support	3.2 (0.5)	3.3 (0.5)	3.3 (0.6)	3.3 (0.6)	3.3 (0.5)	3.3 (0.6)	0.84

Perceived stress	2.5 (0.8)	2.4 (0.8)	2.4 (0.8)	2.4 (0.9)	2.4 (0.9)	2.4 (0.9)	0.78

Health	3.3 (0.9)	3.3 (0.9)	3.3 (0.9)	3.2 (0.9)	3.2 (0.9)	3.2 (0.9)	0.88


**Figure 2:**
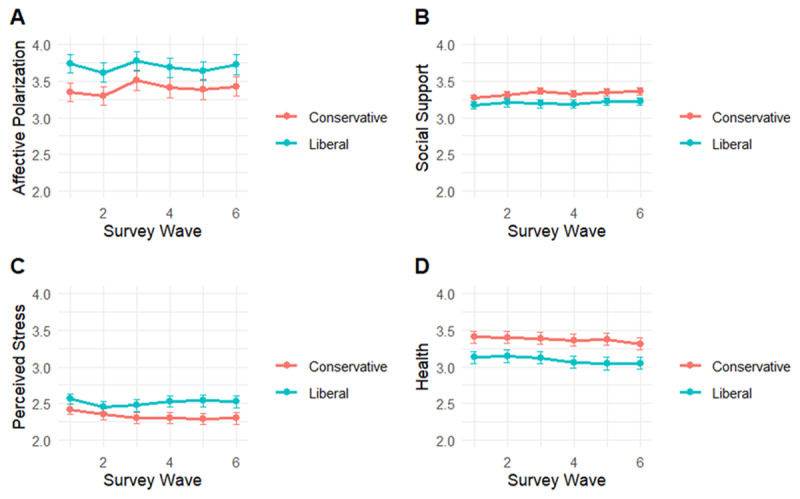
Trends in affective polarization, social support, perceived stress, and self-rated health among liberals (N = 237) and conservatives (N = 233), measured bi-weekly over the course of three months preceding the 2024 US presidential election. Note: Scales do not begin at zero to allow for error bar visibility. Affective polarization values can range from 1–7, social support from 1–4, and perceived stress and health from 1–5. Error bars indicate 95% CIs.

Likewise, when observing trends by political orientation during our study, there is no discernible effect of the encroaching election (Figure 2). However, there are stable average differences between liberals and conservatives. In our sample, liberals were consistently higher in affective polarization (*d* = 0.17–0.27; Panel A) and perceived stress (*d* = 0.13–0.28; Panel C), lower in social support (*d* = 0.18–0.31; Panel B), and had worse self-rated health (*d* = 0.27–0.36; Panel D).

### Pre-registered analyses

All three of our single-indicator RI-CLPMs with constrained autoregressive and cross-lagged effects showed good fit according to our pre-registered criteria (Table 2). Likewise, the chi-square difference tests for these models and their unconstrained versions were non-significant, indicating that imposing the constraints did not result in a significantly worse fit (Table 2).

**Table 2:** Fit statistics for the RI-CLPM models assessing hypotheses H1–H3.


FIT STATISTIC (PRE-REGISTERED FIT CRITERIA)	H1	H2	H3

RMSEA (<.06)	0.05	0.04	0.05

SRMR (<.08)	0.04	0.04	0.03

CFI (>.95)	0.99	0.99	0.99

TLI (>.90)	0.99	0.99	0.99

X^2^	93.67, *p* = .01	95.32, *p* = .008	87.43, p = .03

ΔX^2^ (28)	35.02, *p* = .17	34.66, *p* = .18	32.86, *p* = .24


Note: ΔX^2^ (28) indicates the results of a chi-square difference test with 28 degrees of freedom between the constrained and unconstrained models.

#### H1: Affective polarization and social support

**Figure 3:**
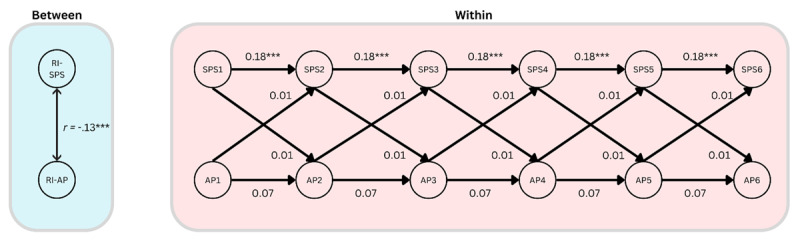
A simplified diagram of the RI-CLPM assessing relationships between affective polarization (AP) and social support (SPS), showing the within-person dynamics over time, and the stable between-person correlation. Note: *** = *p* < .001.

Our first hypothesis, H1, was not supported (Figure 3). There was no significant cross-lagged effect of affective polarization on social support (β = 0.01, *p* = .34, 95% CI [–0.02, 0.04]). Nor was there evidence of a significant effect of social support on affective polarization (β = 0.01, *p* = .96, 95% CI [–0.24, 0.26]). There was a significant autoregressive effect for social support over time (β = 0.18, *p* = .001, 95% CI [0.07, 0.28]), but this was not the case for affective polarization (β = 0.07, *p* = .28, 95% CI [–0.06, 0.20]). However, a significant negative correlation between the random intercepts (*r* = –.13, *p* < .001, 95% CI [–0.20, –0.06]) was found, which is akin to a cross-sectional correlation. This suggests that, at least in the course of our study, there was no evidence of affective polarization negatively impacting social support. However, those who tend to report higher levels of affective polarization do tend to also report lower levels of social support.

#### H2: Affective polarization and perceived stress

**Figure 4:**
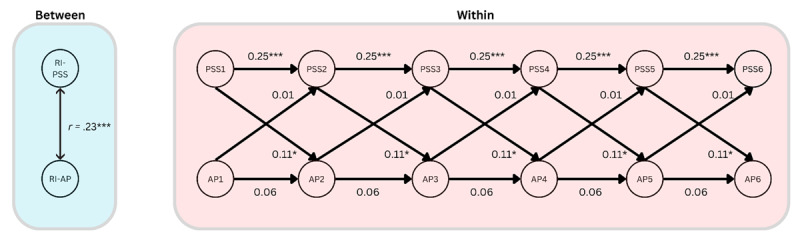
A simplified diagram of the RI-CLPM assessing relationships between affective polarization (AP) and perceived stress (PSS), showing the within-person dynamics over time, and the stable between-person correlation. Note: *** = *p* < .001, * = *p* < .05.

Our second hypothesis, H2, was also not supported (Figure 4). There was no significant positive cross-lagged effect of affective polarization on perceived stress (β = 0.01, *p* = .74, 95%CI [–0.05, 0.07]). There was, however, a small and just significant positive cross-lagged effect of perceived stress on affective polarization (β = 0.11, *p* = .04, 95% CI [0.05, 0.21]), perhaps indicating that a medium-sized (β_standardized_ = 0.08) causal effect runs in the opposite direction. There was a significant autoregressive effect of perceived stress over time (β = 0.25, *p* < .001, 95% CI [0.13, 0.36]), but this was not so for affective polarization (β = 0.07, *p* = .36, 95% CI [–0.07, 0.20]). There was also a significant between-person correlation of the random intercepts (*r* = .23, *p* = < .001, 95% CI [0.13, 0.33]). The results, therefore, indicate that those who report higher levels of affective polarization also tend to report higher levels of perceived stress, and that increases in perceived stress are associated with moderate increases in affective polarization over time.

#### H3: Affective polarization and health

**Figure 5:**
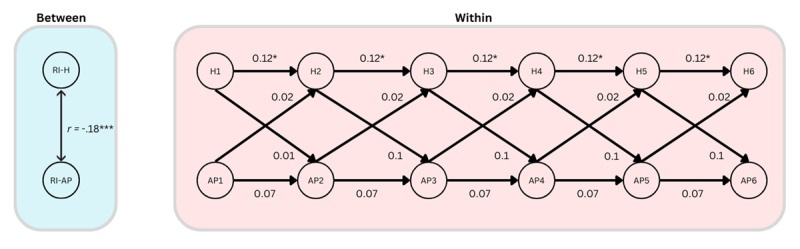
A simplified diagram of the RI-CLPM assessing relationships between affective polarization (AP) and self-reported health (H), showing the within-person dynamics over time, and the stable between-person correlation. Note: *** = *p* < .001, * = *p* < .05.

Our third hypothesis, H3, was also not supported (Figure 5). There was no significant cross-lagged effect of affective polarization on health (β = 0.02, *p* = .32, 95% CI [–0.02, 0.06]). There was also no significant cross-lagged effect of health on affective polarization (β = 0.01, *p* = .85, 95% CI [–0.11, 0.13]). There was a just significant autoregressive effect of health over time (β = 0.12, *p* = 0.04, 95% CI [0.01, 0.23]); this was not so for affective polarization (β = 0.07, *p* = .27, 95% CI [–0.06, 0.20]). There was also a significant negative between-person correlation of the random intercepts (*r* = –.18, *p* = .002, 95% CI [–0.29, –0.07]). This indicates that those who tend to report higher affective polarization also tend to report worse health.

## Discussion

The goal of this study was to investigate potential intrapersonal harms of affective polarization. Specifically, we investigated whether affective polarization negatively impacts one’s social relationships and social support (Chen & Rohla, 2018; Lee, 2021; Smith, Hibbing & Hibbing, 2019), whether affective polarization causes psychological stress (Fraser, et al., 2022; Smith, 2022; Smith, Hibbing & Hibbing, 2019), and whether experiencing affective polarization is ‘like drinking poison’ (Nelson, 2022, p. 01). Our study measured relevant constructs fortnightly over a contentious three-month pre-election period leading up to the 2024 US presidential election, at a time reasonably expected to be characterized by heightened emotions relating to politics.

Contrary to our hypotheses, none of our analyses found evidence suggesting that affective polarization is causally related to the social, mental, and physical harms often ascribed to it. Fluctuations in affective polarization did not predict changes in social support, stress, or self-rated health over time. However, the cross-sectional correlations between our constructs showed the expected relationships based on past observational studies. Higher affective polarization was significantly negatively associated with social support and self-rated health, and significantly positively associated with perceived stress. Therefore, people who, on average, report higher affective polarization tend to also report higher stress, lower social support, and worse health. These associations were small to moderate according to the updated effect size guidelines for the social sciences (Gignac & Szodorai, 2016). We did, however, find tentative evidence that perceived stress is causally related to increased affective polarization. This finding may call into question research which finds that it is primarily anger in response to perceived threats, rather than fear or stress, that contributes to affective polarization (Renström, Bäck & Carroll, 2023).

Not only did we not find sufficient evidence to suggest that one’s own level of affective polarization causes one harm, we also did not see any evidence in trends which would suggest that the election and its attendant ambient polarization had an effect on the constructs of interest. Based on the literature reviewed in this study, one would expect to see a clear increase in affective polarization and stress leading up to the election, and a decrease in social support and health. None of these changes were observed, although there was a stable difference in these constructs by political orientation.

There are multiple potential explanations for our results. When one’s hypotheses are falsified, it is ambiguous whether it is the main ‘target’ hypothesis which has been falsified, or any one of the many auxiliary assumptions (Stanford, 2023). It may simply be that affective polarization does not exert the harmful effects suggested by prior observational research, and that our research correctly identified this fact.

Alternatively, it may be that the effects we aimed to quantify exert themselves on a different timescale and that the length of our time lags was incorrect, causing the effects to be lost in noise. However, according to Dormann & Griffin (2015), longitudinal studies in the social sciences typically use time lags that are far too long, and that ‘shortitudinal’ studies with shorter time lags such as ours are more appropriate. The question of whether our time lags were optimal or not remains open.

### Implications

The present study may have provided evidence that affective polarization is being overly pathologized by researchers spuriously extrapolating to causal conclusions based on cross-sectional associations. These findings represent a significant contribution to the literature on affective polarization and its putative consequences. While the cross-sectional associations between affective polarization and wellbeing, highlighted in past research, were replicated here, the RI-CLPM showed that these may be caused by other confounding variables, as changes in affective polarization did not reflect changes in our wellbeing indicators. This may indicate that the intrapersonal harms associated with affective polarization are exaggerated and adds to a growing literature which questions other harms frequently ascribed to affective polarization (Broockman et al., 2022).

Affective polarization’s intrapersonal harm effects appear increasingly ambiguous, and there may even be *benefits* to affective polarization. From a social identity and intergroup threat theory perspective, affective polarization may reflect a strengthened in-group identification, which may have benefits in that it can enhance self-esteem, provide existential and epistemic security, and foster solidarity among group members (Stephan, Ybarra & Morrison, 2009). Thus, while affective polarization may be somewhat socially alienating and stressful, which may impact health, it may also fulfil other important psychological needs by offering stability, identity, and meaning in opposition to perceived out-group threats. Indeed, some studies have asserted that affective polarization may have other positive outcomes, such as driving political and civic engagement (Iyengar & Krupenkin, 2018), which others have claimed may even improve wellbeing (Nelson, 2022). Additionally, some political theorists assert that a certain level of affective polarization, as part of political *agonism*, may be good for democracy and should not necessarily be discouraged (Mouffe, 1999). Thus, uncritically pathologizing affective polarization may have unexpected negative consequences, and affective polarization may be a more complex phenomenon than previously thought. However, this does not discount the potential interpersonal harm and prejudice that can be driven by affective polarization (Carmines & Nassar, 2022; Iyengar & Westwood, 2015; Lelkes & Westwood, 2017; Westwood et al., 2018), as well as the degrading effect it can have on belief formation, institutional functioning, and democratic norms (Jenke, 2023; Kingzette et al., 2021).

Despite our inability to test the potentially heterogeneous causal relationships among liberals and conservatives, we did replicate here the finding that liberals tend to report being slightly more affectively polarized than conservatives. This may have been due to an anticipated electoral victory for conservatives, though similar findings, largely driven by an asymmetry in desire for social distance among liberals, have also been seen at other times and during other election periods (Casey, Vanman & Barlow, 2023; Cox, 2021; McMurtrie, 2024; Mitchell et al., 2014; Ridge, et al., 2025). However, the differences seen in our results were small, and questions around the presence of asymmetries in political prejudice, polarization, and related constructs such as authoritarianism remain open and contested (Brandt & Crawford, 2020; Ditto et al., 2019; Gidron, Adams & Horne, 2023).

### Limitations

A significant limitation to the present study was the potential lack of statistical power to estimate effects. Our power analysis found that a sample of 500 participants would give 70% power to detect cross-lagged effects of 0.15, or over 80% to detect effect sizes of 0.2. In the context of RI-CLPMs, these are considered relatively large effect sizes (Orth et al., 2024). Because of exclusions and dropouts, we ended up with a final sample of 470 participants. As per our pre-registration, we had hoped to increase that statistical power for H1 and H2 through the use of multiple indicators which facilitate the estimation of measurement error (Mulder, 2023); however, the complexity of the models made them unfeasible. Additionally, beyond the issue of power, our inability to use multiple indicators to model measurement error could have introduced uncertainty into our estimates of causal effects. Therefore, it is possible that the true effects went undetected in our models.

The generalizability of our findings is also limited by the specific research context, both in regard to research timing and cultural context. While the US presents a compelling case for studying affective polarization due to its strong two-party system, its high level of ideological and social sorting, and its extensive partisan media ecosystems, this does mean that our findings may not generalize to other cultures or other political contexts, such as multiparty political systems (Knudsen, 2021; Wagner, 2021). Likewise, while we ran our study in the pre-election period due to the well-established fact that election periods are associated with greater affective polarization (Hernández, Anduiza & Rico, 2021), the proximity of the data collection period to the election may have meant that changes in affective polarization stabilized at the maximum among those participants who are prone to polarization. This may be why our constructs showed a surprising stability over time, and our analysis method designed to track associated *changes* in the constructs found no significant results.

### Future directions

Future studies should aim to replicate the present investigation with a much larger sample size, and/or by increasing the number of survey periods in the study, which increases power by reducing measurement error in the random intercepts, and thus allows for greater sensitivity in estimating within-person effects (Orth et al., 2024).

Future studies could also perform a multiple-group analysis in left- and right-wing participants separately. An a priori data simulation and comprehensive model testing should also be performed to ensure multiple-indicator RI-CLPMs could be run. This would allow researchers to model measurement error and test measurement invariance, which may improve measurement validity and increase statistical power.

Lastly, in the present study, we opted to use complex non-experimental longitudinal analyses which are capable of tentatively making causal claims. This was because, in our view, the pre-election period presented a data collection context in which affective polarization was predicted to be in a state of flux. However, our constructs were relatively stable over the course of our study, so future studies could investigate these findings further by extending the data collection period to continue after an election in order to potentially capture greater variation. This would result in a design closer to an interrupted time series design, which may better allow researchers to quantify the effect of changes in affective polarization on the outcome variables, as affective polarization has shown to peak immediately after an election before declining quickly (Hernández, Anduiza & Rico, 2021). Likewise, experimental designs could be used to investigate these effects with a higher degree of internal validity. However, in the context of affective polarization research, these usually rely on vignette experiments (Huddy & Yair, 2021) or manipulations using trust games (Broockman et al., 2022), both of which are subject to some controversy and questions of validity (Collett & Childs, 2011; Hughes & Huby, 2004; Koppel et al., 2022).
